# Increased Arterial Stiffness in Systemic Lupus Erythematosus (SLE) Patients at Low Risk for Cardiovascular Disease: A Cross-Sectional Controlled Study

**DOI:** 10.1371/journal.pone.0094511

**Published:** 2014-04-10

**Authors:** Karim Sacre, Brigitte Escoubet, Blandine Pasquet, Marie-Paule Chauveheid, Maria-Christina Zennaro, Florence Tubach, Thomas Papo

**Affiliations:** 1 Département de Médecine Interne, Hôpital Bichat, Université Paris Diderot, PRES Sorbonne Paris Cité, Assistance Publique Hôpitaux de Paris, Paris, France; 2 INSERM U1149, Paris, France; 3 Département Hospitalo-Universitaire FIRE (Fibrosis, Inflammation and Remodelling in Renal and Respiratory Diseases), Paris, France; 4 Département de Physiologie, Hôpital Bichat, Université Paris Diderot, PRES Sorbonne Paris Cité, Assistance Publique Hôpitaux de Paris, INSERM U1138, Paris, France; 5 Département d'Epidémiologie et Recherche Clinique, Hôpital Bichat, Université Paris Diderot, PRES Sorbonne Paris Cité, Assistance Publique Hôpitaux de Paris, INSERM CIE 801, Paris, France; 6 Département de Cardiologie, Hôpital Européen Georges Pompidou, Université Paris Descartes, PRES Sorbonne Paris Cité, Assistance Publique-Hôpitaux de Paris, INSERM, UMRS 970, Paris, France; Centro Cardiologico Monzino IRCCS, Italy

## Abstract

Cardiovascular disease (CVD) is a major cause of death in systemic lupus erythematosus (SLE) patients. Although the risk for cardiovascular events in patients with SLE is significant, the absolute number of events per year in any given cohort remains small. Thus, CVD risks stratification in patients with SLE focuses on surrogate markers for atherosclerosis at an early stage, such as reduced elasticity of arteries. Our study was designed to determine whether arterial stiffness is increased in SLE patients at low risk for CVD and analyze the role for traditional and non-traditional CVD risk factors on arterial stiffness in SLE. Carotid-femoral pulse wave velocity (PWV) was prospectively assessed as a measure of arterial stiffness in 41 SLE patients and 35 controls (CTL). Adjustment on age or Framingham score was performed using a logistic regression model. Factors associated with PWV were identified separately in SLE patients and in controls using Pearson's correlation coefficient for univariate analysis and multiple linear regression for multivariate analysis. SLE patients and controls displayed a low 10-year risk for CVD according to Framingham score (1.8±3.6% in SLE vs 1.6±2.8% in CTL, p = 0.46). Pulse wave velocity was, however, higher in SLE patients (7.1±1.6 m/s) as compared to controls (6.3±0.8 m/s; p = 0.01, after Framingham score adjustment) and correlated with internal carotid wall thickness (p = 0.0017). In multivariable analysis, only systolic blood pressure (p = 0.0005) and cumulative dose of glucocorticoids (p = 0.01) were associated with PWV in SLE patients. Interestingly, the link between systolic blood pressure (SBP) and arterial stiffness was also confirmed in SLE patients with normal systolic blood pressure. In conclusion, arterial stiffness is increased in SLE patients despite a low risk for CVD according to Framingham score and is associated with systolic blood pressure and glucocorticoid therapy.

## Introduction

Accelerated atherosclerosis accounts for premature mortality among systemic lupus erythematosus (SLE) patients. Most specialists now consider SLE as an independent risk factor for the development of cardiovascular disease (CVD) [1]–[9].

Although the risk for cardiovascular events in patients with SLE is significant, the absolute number of events per year in any given cohort remains small. Thus, CVD risks stratification in patients with SLE focuses on biological or imaging surrogate markers of subclinical atherosclerosis in attempt to identify atherosclerosis at the earliest stage.

Arterial stiffness reflects changes in mechanical wall properties that occur early at onset of vascular disease and predispose to major cardiovascular disease. Besides peripheral pulse pressure (computed as the difference between systolic and diastolic blood pressure), arterial stiffness is commonly assessed with aortic pulse wave velocity (PWV) measurement [10]. Factors associated with increased PWV include age and long-term effects of traditional cardiovascular risk factors such as blood pressure. In addition, most studies indicate a predictive value of PWV for cardiovascular events and mortality, that is additive to traditional risk factors [11]. Arterial stiffness may be a reliable surrogate marker of cardiovascular risk in patients with SLE [12], [13].

To date, two major studies have evaluated arterial stiffness in SLE patients. The first study concluded that PWV was related to both SLE-related and traditional cardiovascular risk factors. This study however lacked a control population [14]. The second study included SLE or rheumatoid arthritis (RA) patients that were analyzed as a whole [15]. More recently, 2 studies have shown an association between PWV and metabolic syndrome (MetS) in SLE patients [16], [17]. Our study was designed to determine whether carotid-femoral PWV as a measure of arterial stiffness is increased in SLE patients asymptomatic for cardiovascular disease and at low risk for CVD, in comparison to healthy controls and to determine the role for traditional and non-traditional CVD risk factors on arterial stiffness in SLE.

## Patients and Methods

### Study participants

Forty-one consecutive patients with systemic lupus erythematosus (SLE) followed in the department of Internal Medicine, Bichat Hospital, Paris-Diderot University, Paris were enrolled between February and October 2012. All subjects fulfilled at least four of the American College of Rheumatology criteria for SLE [18]. At enrolment, patients were treated with prednisone at a stable dose for at least 3 months. Exclusion criteria consisted of known coronary disease or symptoms suggestive of CVD (angina, arrhythmia, congestive heart failure, stroke, and peripheral arterial disease).

Controls were healthy non-carrier relatives of pseudohypoaldosteronism type 1 patients from a clinical study that aimed to evaluate cardiovascular disease with similar tools (Assistance Publique-Hôpitaux de Paris, NCT00646828). All controls had undergone vascular ultrasound imaging between 2008 and 2011 and none had coronary disease or symptoms suggestive of CVD. The first patient with SLE was considered with respect to age and sex. The clinical trial database was examined to find the first control patient of the same age ±3 years and sex. Matches within the range were found for 35 SLE participants.

Extensive screening for conventional cardiovascular risk factors (age, sex, smoking status, family history of coronary artery disease, body-mass index, waist circumference, hypertension, diabetes mellitus, Framingham score for 10-year risk of heart attack) was undertaken in all cases.

SLE-related data were also analyzed: nephritis, thrombosis, central nervous system involvement, Raynaud's phenomenon, cardiac valvulopathy, pulmonary hypertension and medication including hormonal contraception, statins, hydroxychloroquine, steroid and immunosuppressive treatment.

A family history of coronary artery disease was defined when a first-degree relative had suffered a myocardial infarction or stroke before the age of 55 years in males or before the age of 65 years in females [19]. Height and weight were measured, and the body-mass index was calculated as the weight in kilograms divided by the square of the height in meters. Subjects were considered to have hypertension if they repeatedly had a systolic blood pressure (SBP) of at least 140 mm Hg or a diastolic blood pressure of at least 90 mm Hg. Diabetes mellitus was defined by a persistent finding of fasting hyperglycemia and the need for antidiabetic drug therapy [20]. The risk for cardiovascular events was calculated as the absolute risk within the next 10 years using the Framingham risk equation, which includes age, sex, total cholesterol level, high-density lipoprotein cholesterol level, smoking history, and SBP [19].

Systemic lupus erythematosus disease activity was assessed using the SELENA-SLEDAI score [21], [22].

The diagnosis of antiphospholipid syndrome (APS) was based on a history of venous and/or arterial thromboses or recurrent miscarriages in the presence of aPL antibodies in accordance with published criteria [23].

Blood tests included complete blood count, creatinine, total cholesterol, high-density lipoprotein cholesterol, low-density lipoprotein cholesterol, triglycerides, glycated hemoglobin, homocysteine, 25(OH)-D3 vitamin, lupus serology (anti-DNA antibodies, complement) and antiphospholipid antibodies (anticardiolipin and anti-β2GP1 antibodies, lupus anticoagulant). Urine sample was assayed for creatinine, red blood cells and proteins.

### Vascular assessment

Vascular ultrasound study was performed in the context of care, in a temperature-controlled room after a 15 min rest (Vivid 7, General Electric, Horten, Norway). All subjects had fastened for at least 12 hours before PWV evaluation. A single investigator conducted vascular measurements in controls and SLE patients. All data were analysed offline (EchoPAC, General Electric Ultrasound). Aortic pulse wave velocity (PWV) was measured by the carotid to femoral method [24]. The time delay between ECG R wave and the Doppler flow onset was measured at right common carotid artery and common femoral artery and the difference was computed (t). D is the body surface distance between the 2 measurement points minus the distance from the suprasternal notch to the carotid measurement point. PWV was computed as D/t. Internal carotid wall thickness (ICWT) was measured at the carotid bulb level at end diastole, as gated on ECG. Right and left values were averaged for each patient. Carotid plaques were defined as thickness greater than 2 mm.

### Statistical analysis

Continuous variables are expressed as mean (SD) or median (IQR). Categorical variables are expressed as frequencies and percentages. Data were compared between SLE patients and controls using Chi2 test (or Fisher) for dichotomous variables and Student test (or Wilcoxon non-normally distributed) for continuous variables. The two-way ANOVA test was performed when specified. Arterial stiffness was compared between SLE patients and controls using the Wilcoxon rank-sum test. Adjustment on age or Framingham score was performed with a logistic regression model. Factors associated to PWV were identified separately in SLE patients and in controls by use of the Pearson's correlation coefficient (univariate analysis) and multiple linear regression (multivariate analysis). Variables correlated to PWV in univariate analysis with a p-value below 10% were considered for the multivariate model. Variable selection relied on the AIC index.

### Ethics statement

Our study is a human non-interventional study where 1-subjects were not assigned to treatment, 2-subjects were assigned to a diagnosis strategy within current practice (i.e. vascular ultrasound), 3-study involved products with a marketing authorization that are prescribed in the usual manner and used in accordance with French agencies authorizations (i.e. vascular ultrasound), 4-epidemiological methods were used to analyze the data, and 5- information used in the study were collected for clinical care.

According to the Public Health French Law (art L 1121-1-1, art L 1121-1-2), approval from institutional review board and written consent are not required for human non-interventional studies. For ethical consideration, patients were however informed that data that was collected in medical records might be used for research study in accordance to privacy rule. Written informed consent was obtained from each patient and the study protocol conforms to the ethical guidelines of the 1975 Declaration of Helsinki. Our study involves personal health data and has been authorized by the Commission nationale de l'informatique et des libertés (CNIL) (declaration number 1685680 v 0)

## Results

### Characteristics of SLE patients and controls

Forty-one SLE patients and 35 controls (CTL) were studied. The mean age of SLE subjects was 39±10 years (37±8 in controls) and 34 (82.9%) were female (80% in controls). Forty patients (97.5%) received long-term glucocorticoids and 27 (65.9%) still used prednisone at a mean daily dose of 9±3 mg (range: 5–17) at study time. Twenty-eight (68.3%) had received immunosuppressive or immunomodulatory drugs at some point during follow-up. SLE patients were treated with angiotensin converting enzyme inhibitor or angiotensin receptor antagonist (n = 18), beta blocker (n = 3) or calcium channel blocker (n = 1). No control subject was receiving anti-hypertensive drugs at PWV measurement.

Age, sex, tobacco use, hypertension, diabetes, BMI, LDL-cholesterol level, or absolute risk of cardiovascular events occurring within the next 10 years according to the Framingham score were not statistically different between SLE patients and controls. LDL-cholesterol level was lower in SLE patients as compared to controls (0.92±0.3 vs 1.2±0.3 g/l, p = 0.001). Only one SLE subject (2.4%) had a family history of CVD.

Among non-traditional CVD risk factors in SLE patients, waist circumference was above 88 cm (ranging from 90 to 150 cm) in 64.7% (22/34) of women and above 102 cm in 14.3% (1/7) of men. Sixteen (39%) patients had a blood homocysteine/creatinine ratio above the normal value of 0.17 (range: 0.18 to 0.42). Twenty-four (61%) had a vitamin D deficiency assessed by a blood level of 25(OH)-D3 vitamin below 30 ng/ml (range: 5 to 28.1).

Clinical characteristics of SLE patients and controls are shown in detail in Table 1.

**Table 1:** Characteristics of SLE (systemic lupus erythematosus) and controls subjects.

	SLE patients (n = 41)	Controls (n = 35)	p
Female Sex, n (%)	34 (82.9)	28 (80)	0.77
Age, years	39 (±10) (21–62)	37 (±8) (23–59)	0.21
Familial History of CAD, n (%)	1 (2.4)	NA	
Smoker current, n (%)	14 (34.1)	12 (34.3)	1
Hypertension, n (%)	12 (29.2)	8 (22.9)	0.61
Diabetes, n (%)	1 (2.4)	1 (2.8)	1
BMI, kg/m^2^	25.5 (±5.4) (18–44)	26.7 (±6.6) (19–41)	0.69
Waist circumference, cm	93.4 (±15) (72–150)	94.9 (±15.5) (78–131)	0.63
Systolic Blood Pressure, mmHg	130+18 (96–169)	127+11 (115–150)	0.37
LDL-Cholesterol, g/l	0.92 (±0.3) (0.36–1.53)	1.2 (±0.3) (0.62–1.73)	0.001
HbA1c, %	5.4 (±0.5) (4.4–6.7)	5.4 (±0.4) (4.7–6.1)	0.39
10-year risk of heart attack, %	1.8 (±3.6) (0.5–23)	1.6 (±2.8) (0.5–16)	0.53
Homocysteine/creatinine ratio	0.18 (±0.07) (0.06–0.42)		
25(OH)-D3 vitamin, ng/ml	23.4 (±11.5) (5–41.7)		
GFR, ml/mn/1.73 m^2^	84.9 (±34.6) (16.3–168.4)	102 (±21) (63–164)	0.002
Proteinuria/Creatininuria, mg/mmol	85.4 (±215) (5–1188)		
Lymphocytes, G/l	1.4 (±0.6) (0.3–3.4)		
Duration of SLE disease, y	13 (±7.3) (1–37)		
SELENA SLEDAI score	2.1 (±2.8) (0–13)		
Lupus nephritis, n (%)	27 (65.8)		
LA antibodies, n (%)	7 (17)		
aCL antibodies, n (%)	12 (29.3)		
anti-B2GP1 antibodies, n (%)	2 (4.9)		
APS, n (%)	5 (12.2)		
Cumulative years of steroid treatment	10.5 (±7) (0–26)		
Cumulative dose of steroid treatment, g	42.3 (±29.2) (0–132)		
Antiplatelet treatment, n (%)	7 (17.1)		
Anticoagulant treatment, n (%)	6 (14.6)		
Hormonal contraception, n (%)	12/34 (35.3)		
Statin, n (%)	10 (24.4)	1 (2.8)	0.009
Hydroxychloroquine, n (%)	41 (100)		
Other therapy, n (%)	28 (68.3)		

CAD, coronary artery disease; BMI, body mass index; SELENA, safety of estrogens in lupus erythematosus national assessment; SLEDAI, systemic lupus erythematosus disease activity index; LA, lupus anticoagulant; aCL, anti-cardiolipin antibody; β2GP1, β2-Glycoprotein 1; APS, antiphospholipid syndrome.

10-year risk of heart attack was calculated using the Framingham equation.

Normal value for homocysteine/creatinine ratio was below 0.17.

Normal value for 25(OH)-D3 vitamin was above 30 ng/ml (nanogram per liter)

GRF, glomerular rate filtration, calculated with the Modification of Diet in Renal Disease (MDRD) equation and expressed in milliliters per minute per 1.73 square meters of body surface area

Hormonal contraception was progestin-only pill in all cases.

Other therapy included cyclophosphamide (n = 24), azathioprine (n = 14), mycophenolatemofetil (n = 11), methotrexate (n = 3), rituximab (n = 1), or intravenous immunoglobulin (n = 1)

cm, centimeter; g/l, gram per liter; g, gram; millimeter of mercure, mmHg

Results are shown as mean and (SD), and (range)

### Arterial stiffness and carotid wall thickness are higher in SLE patients

**Figure 1:**
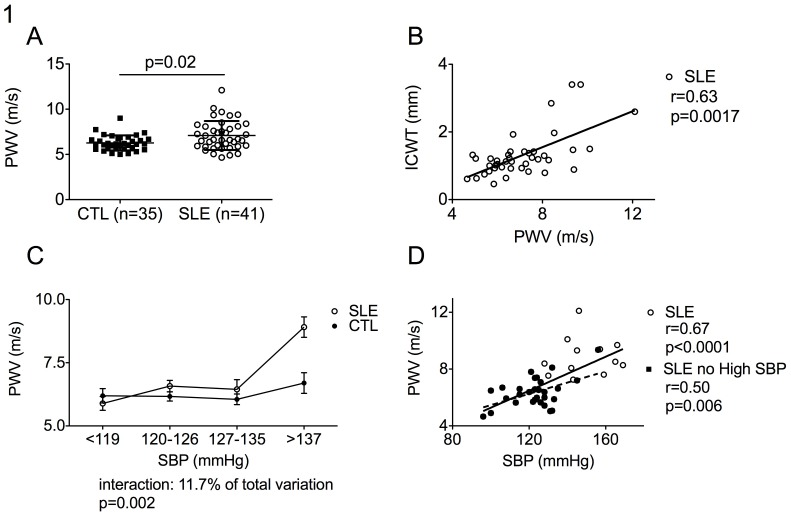
Pulse Wave Velocity (PWV) is high in SLE patients and correlates with systolic blood pressure. (A) Pulse wave velocity (PWV) are higher in SLE patients (SLE, n = 41, right) as compared to controls (CTL, n = 35, left) and (B) correlated with internal carotid wall thickness (ICWT). (C) SLE patients and controls were stratified according to systolic blood pressure quartiles (SBP <119; 120–126, >127–135, >137 mmHg). Two-ways ANOVA showed a significant independent interaction of SBP and group (SLE, CTL) on PWV. (D) PWV correlated with systolic blood pressure (SBP) in SLE subjects with (white circles) and without (black circles) high systolic blood pressure. Plain and dashed lines represented linear regression in SLE subjects with and without high systolic blood pressure (SBP), respectively. p was calculated after age adjustment. m/s refers to meter per second; mm, to millimeter; r, to linear correlation coefficient

Pulse wave velocity (PWV) was higher in SLE patients as compared to controls as shown in Figure 1A (7.1±1.6 vs 6.3±0.8 m/s; p = 0.02 after age adjustment; p = 0.01 after Framingham score adjustment). In the same line, internal carotid wall thickness (ICWT) was higher in SLE patients (ICWT: 1.31±0.67 vs 0.93±0.25 mm; p = 0.007 after age adjustment; p = 0.003 after Framingham score adjustment) and eight (19.5%) SLE patients, but only one control (2.8%) (p = 0.03), displayed a carotid atherosclerotic plaque as defined as a local wall thickening greater than 2 mm. Moreover in SLE patients, PWV significantly correlated with ICWT (r = 0.63, 95% CI: 0.4–0.79, p = 0.0017 after age adjustement) (Figure 1B).

### Risk factors for arterial stiffness: univariate analysis

In SLE patients, PWV was shown to correlate positively with age, systolic blood pressure (SBP), cumulative dose of glucocorticoids and glomerular filtration rate (GFR). No statistical relationship was found between PWV and Framingham score, BMI, blood levels of LDL-cholesterol, homocystein, 25(OH)-D3 vitamin, lymphocytes count, anti-dsDNA antibodies level, current dose of steroids, C3 level, anti-β2GP1 or anticardiolipine antibodies levels and SELENA SLEDAI score. Correlations between PWV and glycated hemoglobin or duration of SLE disease failed to reach statistical significance (Table 2). In controls, PWV only correlated with age and Framingham score (Table 3).

**Table 2:** Univariate analysis of risk factors for arterial stiffness in SLE subjects.

	Number of subjects	Mean PWV (SD)	Median PWV (IQR)	p
Sex				0.60
Male	7	4.9 (9.3)	6.8 (1.5)	
Female	34	4.7 (12.1)	7.2 (1.6)	
Smoker current				0.54
No	27	4.7 (10.1)	7.0 (1.5)	
Yes	14	4.9 (12.1)	7.3 (1.9)	
Diabetes				0.78
No	40	4.7 (12.1)	7.1 (1.6)	
Yes	1	7.5 (7.5)	7.5 (.)	
High blood pressure				<0.0001
No	29	4.7 (9.4)	6.4 (1.0)	
Yes	12	5.7 (12.1)	8.5 (1.6)	
Statin				0.85
No	31	4.7 (12.1)	7.1 (1.5)	
Yes	10	4.9 (10.1)	7.0 (1.9)	
Lupus Nephritis				0.40
No	14	4.9 (10.1)	6.8 (1.5)	
Yes	27	4.7 (12.1)	7.2 (1.7)	
Familial History of CAD				0.66
No	40	4.7 (12.1)	7.1 (1.6)	
Yes	1	7.8 (7.8)	7.8 (.)	
APS				0.54
No	36	4.7 (10.1)	7.0 (1.4)	
Yes	5	4.9 (12.1)	8.0 (2.8)	

	Linear correlation coefficient	95% CI	P
Age	0.49	0.21 to 0.69	0.001
Systolic blood pressure	0.67	0.44 to 0.81	<0.0001
GFR	−0.52	−0.71 to −0.25	0.0003
BMI	0.13	−0.18 to 0.42	0.41
10-year risk of heart attack	0.23	−0.08 to 0.5	0.14
LDL-Cholesterol	0.12	−0.20 to 0.41	0.47
Waist circumference	0.08	−0.24 to 0.38	0.62
HbA1c	0.26	−0.05 to 0.52	0.10
Cumulative steroid dose	0.38	0.07 to 0.61	0.01
Homocysteine/creatinine ratio	−0.23	−0.5 to 0.09	0.16
25(OH)-D3 vitamin	0.03	−0.29 to 0.34	0.84
Duration of SLE disease	0.26	−0.05 to 0.53	0.10
Proteinuria/Creatininuria	0.15	−0.17 to 0.44	0.37
SELENA SLEDAI score	−0.18	−0.46 to 0.14	0.27
C3	0.24	−0.04 to 0.50	0.09

CAD, coronary artery disease; BMI, body mass index; SELENA, safety of estrogens in lupus erythematosus national assessment; SLEDAI, systemic lupus erythematosus disease activity index; APS, antiphospholipid syndrome.

10-year risk of heart attack was calculated using the Framingham equation.

Normal value for homocysteine/creatinine ratio was below 0.17.

Normal value for 25(OH)-D3 vitamin was above 30 ng/ml (nanogram per liter)

GRF, glomerular rate filtration, calculated with the Modification of Diet in Renal Disease (MDRD) equation and expressed in milliliters per minute per 1.73 square meters of body surface area

PWV, pulse wave velocity is expressed in meter per second

IQR, interquartile range

SD, standard deviation

**Table 3:** Univariate analysis of risk factors for arterial stiffness in Control subjects.

	Number of subjects	Mean PWV (SD)	Median PWV (IQR)	p
Sex				0.73
Male	7	5.5 (7.7)	6.4 (0.8)	
Female	28	5.0/9.0	6.2 (0.9)	
Smoker current				0.51
No	23	5.0/9.0	6.3 (0.8)	
Yes	12	5.1/7.7	6.1 (0.8)	
Diabetes				0.69
No	1	6.6/6.6	6.6 (.)	
Yes	34	5.0/9.0	6.3 (0.8)	
High blood pressure				0.33
No	27	5.0/7.7	6.2 (0.7)	
Yes	8	5.5/9.0	6.5 (1.1)	

	Linear correlation coefficient	95% CI	p
Age	0.48	0.17 to 0.70	0.003
Systolic blood pressure	0.17	−0.17 to 0.48	0.32
GFR	−0.15	−0.46 to 0.19	0.38
BMI	0.04	−0.30 to 0.37	0.82
10-year risk of heart attack	0.37	0.03 to 0.62	0.03
LDL-Cholesterol	0.30	−0.04 to 0.57	0.08
Waist circumference	0.04	−0.30 to 0.37	0.82
HbA1c	0.05	−0.29 to 0.38	0.78

BMI, body mass index;

10-year risk of heart attack was calculated using the Framingham equation

GRF, glomerular rate filtration, calculated with the Modification of Diet in Renal Disease (MDRD) equation and expressed in milliliters per minute per 1.73 square meters of body surface area

PWV, pulse wave velocity is expressed in meter per second

IQR, interquartile range

SD, standard deviation

The frequency of subjects with high blood pressure (29.2% of SLE patients vs 22.9% of controls, p = 0.61) and the mean SBP at study time (130±18 in SLE vs 127±11 in CTL, p = 0.37) did not significantly differ between groups. Interestingly, after stratification according to SBP quartiles, the correlation between SBP and PWV for a given level of SBP appeared to be higher in SLE patients as compared to controls. Indeed the interaction between SBP and SLE status was accounting for 11.7% of the observed variance (p = 0.002) (Figure 1C). Moreover, the correlation between PWV and SBP was observed even in SLE patients without high systolic blood pressure (r = 0.50, p = 0.006) (Figure 1D).

The proportion of SLE patients with high blood pressure and the mean SBP at study time did not significantly differ between patients that were currently receiving or not glucocorticoid (33.3% (9/27) vs 35.7% (5/14), p = 1.00 and 130±17 (range: 96–169) vs 132±19 (108–165) mmHg, p = 0.67; respectively).

Twenty-seven (65.8%) SLE patients had experienced lupus nephritis in the past, mostly (20/27, 74%) class IV diffuse nephritis according to International Society of Nephrology/Renal Pathology Society (ISN/RPS) classification of lupus nephritis [25]. No SLE patient displayed active lupus nephritis at study time. No history of renal disease was reported in controls. The glomerular filtration rate (GFR), calculated with the Modification of Diet in Renal Disease (MDRD) equation, was below 60 ml/mn/1.73 m^2^ (range: 16.3 to 55.6) in 8 (19.5%) SLE patients. All control subjects displayed a GFR above 60 ml/mn/1.73 m^2^ (p = 0.01, as compared to SLE). Moreover, the mean GFR was significantly higher in controls (102±21 ml/mn/1.73 m^2^) as compared to SLE patients (84.9±34.6, p<0.05), especially as compared to SLE subjects with history of lupus nephritis (74.5±30.6, p<0.005).

The mean protein urinary output, assessed by measuring the morning urinary protein/creatinine ratio, was 84.5 (±215) mg/mmol in SLE patients and 13 (31%) patients had a ratio above 30 mg/mmol (range: 41.3 to 1188). No correlation was found between PWV and urinary protein/creatinine ratio in SLE patients (p = 0.36).

### Risk factors for arterial stiffness: multivariate analysis

In the multivariate analysis (Table 4), both systolic blood pressure (p = 0.0005) and cumulative prednisone intake (p = 0.01) were associated with arterial stiffness in SLE patients. The association between age and PWV failed to reach statistical significance (p = 0.08). In controls, only age was associated with PWV (p = 0.005)

**Table 4:** Multivariate analysis of risk factors for arterial stiffness.

SLE subjects				
	Linear correlation coefficient	Unstandardized betas	95% CI	p
Age	0.49	0.03	0.21 to 0.69	0.08
Systolic blood pressure	0.67	0.04	0.44 to 0.81	0.0005
GFR	0.52	−0.008	0.71 to 0.25	0.17
Cumulative steroid dose	0.38	0.015	0.07 to 0.61	0.01

Controls				
	Linear correlation coefficient	Unstandardized betas	95% CI	p
Age	0.48	0.04	0.17 to 0.70	0.005
LDL-Cholesterol	0.30	0.69	0.04 to 0.57	0.10

GRF, glomerular rate filtration, calculated with the Modification of Diet in Renal Disease (MDRD) equation and expressed in milliliters per minute per 1.73 square meters of body surface area

## Discussion

Cardiovascular diseases (CVD) are a major cause of morbidity and mortality in systemic lupus erythematosus (SLE) [1]–[6]. The pathogenesis of CVD associated with SLE is unknown. Our study shows that, despite a low cardiovascular risk according to Framingham score, SLE patients display increased arterial stiffness as compared to controls. Systolic blood pressure (SBP) and glucorticoid treatment are the main contributors to this phenomenon. Moreover we observe that, in SLE patients, increased arterial stiffness parallels with subclinical atherosclerosis assessed by internal carotid wall thickness (ICWT) measurement.

Arterial stiffness is a consequence of arterial wall thickening and early changes in mechanical properties of the vascular wall. Previous research has found a strong association between hypertension and arterial stiffness in general population [26], [27]. Moreover, hypertension was shown to be associated with an increased frequency of cardiovascular events (CVE) in SLE patients [28]. Interestingly, in our study, SBP correlated with arterial stiffness even in SLE patients with normal blood pressure. Moreover, at a given level of SBP, the correlation between systolic blood pressure and arterial stiffness, was stronger in SLE patients as compared to controls.

Hypercholesterolemia was reported to be the best predictor of CVE in SLE patients [28]. We did not confirm such correlation between hypercholesterolemia and arterial stiffness in SLE patients. However, treatment with statins was started when the LDL-cholesterol was >1 g/l according to current guidelines in our SLE patients [29]. Statin treatment explains the fact that level of LDL-cholesterol is lower in SLE patients as compared to controls.

In previous studies, SLE-specific variables appeared to be independently associated with increased PWV [14], [17]. Although we may argue that the cumulative dose of steroid taken is related to a more severe SLE disease, we do not observe significant association between SLE-related variables - such as disease activity score or duration of SLE disease - and arterial stiffness.

Among non-traditional risk factors for atherosclerosis in SLE patients, the role played by glucocorticoids, a first line treatment for lupus, remains a matter of debate. The estimated 2-year coronary risk for a patient treated with an average dosage of 30 mg/day of prednisone for 1 year, is approximately 60% higher than it would be for a patient with the same SLE activity level and similar risk factors receiving no steroids [30]. On the other hand, since inflammation is implicated in atherosclerosis, low dose corticosteroids may exert a beneficial anti-inflammatory action [31]. Subclinical atherosclerosis in SLE has indeed been correlated with both a low dose of corticosteroids and less immunosuppressive drugs [5]. In contrast, our study suggests that, even in subjects at low risk for CVD according to Framingham score, cumulative prednisone treatment contributes to arterial stiffness and may play a role in accelerated atherosclerosis in SLE. An association between PWV and metabolic syndrome has recently been shown in two cross-sectional studies in SLE patients [16], [17]. Consistent with our data, metabolic syndrome in SLE patients was also associated with a higher daily prednisolone dose [32].

Our study has several limitations. First, sample size is small and our work may lack statistical power to show the effect of SLE-specific factors on arterial stiffness. Second, causation cannot be concluded from a cross-sectional study. Eventually, biases cannot be excluded in the selection of healthy controls.

In conclusion, our results support the fact that both systolic blood pressure and glucocorticoid therapy are major contributors to premature arterial stiffness in SLE patients.
